# A Personal History in Parasitic Weeds and Their Control

**DOI:** 10.3390/plants10112249

**Published:** 2021-10-21

**Authors:** Chris Parker

**Affiliations:** Independent Researcher, 6 Royal York Crescent, Bristol BS8 4JZ, UK; chrisparker5@compuserve.com

**Keywords:** parasitic weeds, *Striga*, *Orobanche*, *Phelipanche*

## Abstract

This invited paper summarises a career in which I became increasingly involved in research and related activities on *Striga* and other parasitic weeds. It also presents a personal view of the present status of parasitic weed problems and their control.

## 1. Introduction

Prior to 1950, the most notable achievement in parasitic weed control had been the discovery in the early 20th century, of immunity to *Orobanche cumana* in sunflower. In their excellent review, Molinero-Ruiz et al. [1] describe how *O. cumana* (then known as *O. cernua*) was first recognised as a problem in sunflowers in the Black Sea region of Russia in 1866, and by the end of the century had spread to Moldova and Romania. Pustovoit [2] describes how, in 1912, a resistance breeding programme was initiated. Cubero [3,4], in other valuable reviews, describes how, by 1916, a range of material had been developed with complete resistance and by 1925, 95% of the sunflower crop grown in Pustovoit’s region was based on these resistant lines. In the following three years, however, the resistance failed due to the emergence of different races of the parasite, the original being designated race A and the more virulent, race B. In due course, new varieties were required with resistance to races C, D, E, F, G. and H.

Other parasitic weeds, notably other *Orobanche*, *Phelipanche* and *Striga* problems have proved more intractable. The earliest reports of work on *Striga* as a weed problem are for *S. asiatica* from South Africa where Pearson [5] showed the benefit of nitrogen fertilization and Saunders from 1926 [6] onwards through to 1942, conducted a wide range of studies on biology and control, proposing a number of agronomic approaches, and most importantly began the process of breeding and selection for resistance in sorghum, leading to release of the variety Radar. Timson worked on serious infestations of *S. asiatica* in maize in Rhodesia (Zimbabwe) from 1929 onwards. He promoted catch-cropping with sudan grass, (*Sorghum x drummondii*) together with phosphate fertilizer and crop rotation leading to over 3-fold yield increases in maize over a four-year period [7].

The problem from *S. hermonthica* in the northern half of Africa did not appear to be recognised until somewhat later. Presumably, there was less pressure on land for arable cropping, and it was traditionally suppressed by shifting cultivation. It was first recorded as a problem in maize in Kenya in 1928, and later in sorghum in Sudan, and in rice in Senegal. One of the first to achieve some success in control was Doggett [8], working in Tanzania from 1952 to 1954, who developed a number of resistant sorghum varieties, including Dobbs and others which provided the basis for subsequent work on resistance by the International Crops Research Institute in the Semi-Arid Tropics (ICRISAT) and others.

We have all this early information thanks to the extraordinarily detailed abstracts compiled by McGrath et al., [9] and published by the US Department of Agriculture (USDA) following the discovery of widespread infestation of maize by *S. asiatica* (first reported as *S. lutea*) in the Carolinas of USA, after what must have been a decade unidentified. The fear was that it could spread to the corn belt of the mid-West. This led to the establishment of the Witchweed Lab in Whiteville, North Carolina in 1965, headed by the late Bob Eplee, and a quarantine campaign and series of valuable studies on the biology and control of this species. These included methods for monitoring infestations by separation of seeds from the soil, the use of herbicide to prevent new seed production, and the use of ethylene to stimulate suicidal germination. Much of this work was summarised in the Weed Science Society of America (WSSA) publication by Sand et al. [10]. Wide-scale quarantine restrictions were finally lifted in 2009 and by 2015, $250 million later, it was finally reduced to just over 1000 acres [11].

Beyond the 1950s, major publications across the years have included Job Kuijt’s masterly volume on The Biology of Parasitic Flowering Plants, the first to provide an overall survey of the subject [12]. Since then, some of the further major publications have included: Musselman, [13], Visser [14], Press and Graves [15], Joel et al. [16], Heide-Jorgenson [17] Joel et al. [18] and many others have appeared each year.

## 2. Our studies in the UK

**Figure 1:**
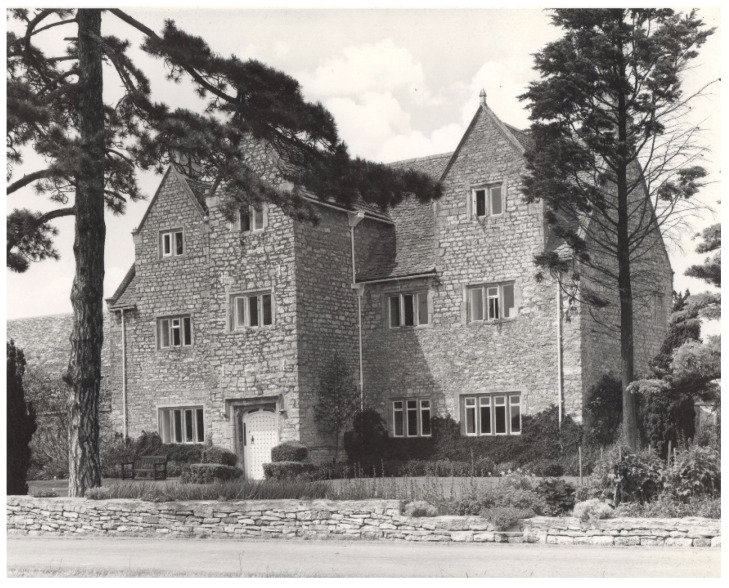
Weed Research Organisation, Begbroke Hill, Oxford, closed 1985. There were modern labs behind.

My introduction to parasitic weeds was in the 1950s when I was working for a chemical company in South Africa evaluating their herbicides on field crops, and saw *S. asiatica* for the first time in maize. I did no work on it then but on return to the Agricultural Research Council (ARC) Unit of Experimental Agronomy in Oxford, UK in 1959, I inherited a programme of herbicide evaluation, partly funded by the UK Ministry of Overseas Development (later the Overseas Development Organisation) which already involved work on *S. hermonthica.* From then on, I gradually became more specialised in parasitic weeds, though for most of my career continuing with projects on biology and control of other weeds. There, and subsequently at the ARC Weed Research Organisation (WRO) (Figure 1) to which I was transferred in 1966, we spent many years delving into *Striga* spp., their biology and control, in lab and glasshouse. We screened innumerable herbicides but found none sufficiently selective. In conjunction with the International Crops Research Institute for the Semi-Arid Tropics (ICRISAT), we screened hundreds of sorghum varieties for their stimulant exudation, helping to lead to the standard low-stimulant variety SRN49 [19]. We also found good resistance in semi-wild, ‘shibra’ millet, but it did not prove practical to exploit this resistance [20].

We developed a polythene bag technique for testing the effects of nutrients and other studies, allowing ready observation of parasite development [21]. We enjoyed demonstrating the profound inhibitory effect of *Striga* extremely early after host attachment, such that, at 4 weeks, less than 1 mg of *S. hermonthica* seedlings a few mm long could cause 400 mg reduction in the total weight of the host, and at 5 weeks, 13.5 mg caused a total weight loss of 960 mg. Furthermore, the shoot is disproportionately affected as a result of a significant shift in root:shoot balance [22]. We confirmed the effect of nitrogen in reducing infection and of potassium in stimulating it but somehow failed to show the effect of phosphorus in reducing it, now well confirmed by others. It has been shown possible to exploit this beneficial effect of P very economically by ‘micro-dosing’ [23].

We looked at a stage in parasite infection that has been generally neglected—the role of chemotropism—and found significant evidence for its role in the orientation of *Striga* seedlings towards the host root prior to attachment [24].

A significant date in the history of *Striga* research is when Cook et al. [25] first described the structure of a strigolactone—strigol, from the roots of cotton. There was soon interest in synthesising simpler analogues and Prof. Alan Johnson, director of an ARC research unit at the University of Sussex set a graduate student to the task. He did not immediately have a means of assaying products for their activity and approached Prof Geoffrey Blackman, director of our sister unit at Oxford University (at an ARC dinner) for advice. He said we should be able to help and within a week, we had the first results showing that the very first compounds to be produced had high activity on pre-conditioned *S. hermonthica* seeds [26]. Hence the student Gerald Rosebery achieved instant immortality through his initials, in the form, initially, of GR2, GR3, GR5, GR7, and eventually, in the standard GR24.

Lytton Musselman and I first met in 1973 (at the Malta meeting—see below) and his friendship and support have been pivotal to me ever since. He joined me for an extremely productive sabbatical at WRO in 1980 and we conducted a range of joint studies, on *Striga, Phelipanche* and *Orobanche* spp., covering their host ranges, specificity, pollination and seed morphology. Some of the first electron micrographs of the seeds of *Striga* species showed clear specific variations [27]. We also confirmed the narrow host specificity of *S. gesnerioides* strains parasitizing wild species [28]. and the relative lack of host specificity in *O. crenata*, *Phelipanche ramosa* and *P. aegyptiaca*, while *O. minor* showed distinct strains [29]. A pollination study confirmed the strict autogamy of *S. asiatica, S. gesnerioides* and *S. forbesii,* also *O. cernua* and *O. minor*, while *S. hermonthica* is dependant on out-crossing. *Orobanche crenata* and *P. aegyptiaca* proved mainly allogamous but facultatively autogamous [30,31].

In 1985 I visited Charlie Riches in Botswana whose work there included the problem of *Alectra vogelii* in cowpea. By then he had identified a number of cowpea landraces with resistance to *Alectra.* I returned to UK with samples of ten of these and in a simple screening to look for possible co-resistance to *S. gesnerioides*, nine out of the ten showed no resistance but B.301 had apparent immunity to both species. In 1984, the variety Suvita-2 had been shown to resist *S. gesnerioides* in Burkina Faso, but it was soon learnt that this line and another, 58–57 were not resistant in Mali, Niger or Nigeria [32]. Our further work with B.301 showed that it was immune to the races from all these countries and from Cameroon [33]. Only in 1993 was it found to be overcome by the ‘hyper-virulent’ Zakpota race from southern Benin. More recent work is ably reviewed in the paper by Botanga and Timko [34] establishing that there have been other lines identified by the International Institute of Tropical Agriculture (IITA) with broad-spectrum resistance, including to the Zakpota race, but B.301 continued to be valuable to IITA in the development of cowpea lines with dual resistance to both *Striga* and *Alectra.* A study of the host specificity of *A. vogelii* showed there to be at least 3 strains varying in their virulence on cowpea, groundnut and bambara [35]. One further study showed that populations of *A. vogelii* are different in West Africa and southern Africa [36].

One of my final research projects involved a study of the involvement of ethylene in the germination of *Striga*. Ethylene had been used as an important means of stimulating suicidal germination and hence reducing *S. asiatica* seed in the soil in the USA. We wondered to what extent ethylene was involved in the activity of the strigolactones. We confirmed that GR24 greatly stimulated evolution of ethylene in the seeds and significantly increased germination of *S. hermonthica* (the increase was prevented by the ethylene inhibitor, norbornadiene) but was not essential to GR24 activity [37].

## 3. International Parasitic Plant Meetings

**Figure 2:**
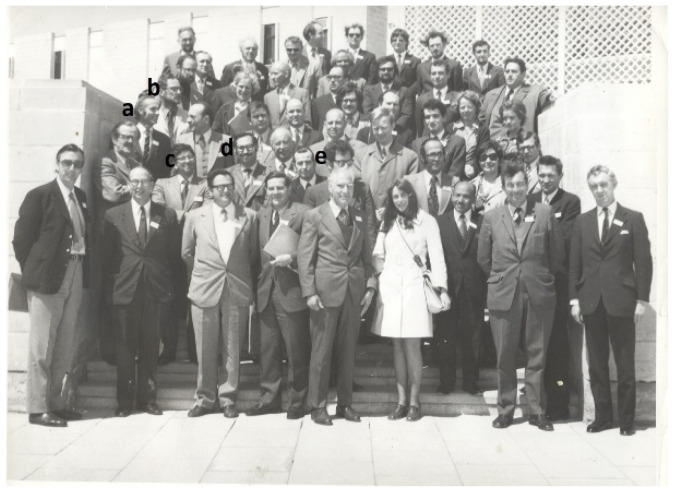
The Malta participants 1973. (**a**) Chris P; (**b**) Job Kuijt; (**c**) Jose Cubero (**d**) Abed Saghir; (**e**) Lytton M.

In the early 1970s, Dr Abed Saghir of the American University of Beirut and I established the European Weed Research Council (EWRC) Parasitic Weeds Research Group. We located just over 100 workers on parasitic weeds in 36 countries in Europe and beyond and invited them to attend a Symposium on Parasitic Weeds in Malta in April 1973 (Figure 2). About 50 attended including Lytton Musselman—also Jose Cubero and Job Kuijt but not many others who are still active. The Proceedings were published by EWRC. This meeting arose out of a UK-funded project on *Orobanche crenata* in Malta in faba beans, led by the late Prof Bill Edwards, whom I had visited in 1970.

**Figure 3:**
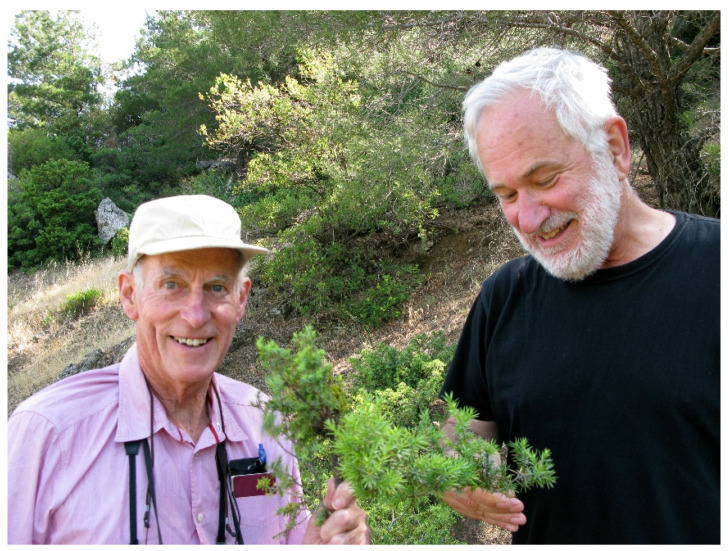
Chris P. and Lytton M. hunting for *Arceuthobium oxycedri* on Mt Sypilos in Turkey 2009.

After 1973, the EWRC Parasitic Weeds Research Group was taken over by the newly formed European Weed Research Society but it was difficult to give adequate emphasis to *Striga* in a European context, so after a brief divorce, it re-formed in 1979 as the International Parasitic Seed Plant Research Group, which later still was taken under the wing of the International Parasitic Plant Society (IPPS)**.** Lytton Musselman in due course arranged a Second International meeting in Raleigh, North Carolina in 1979. This meeting has been followed by further major meetings around the world every few years—the next, the 16th, to be held in Kenya 2022. The earlier meetings involved the preparation of proceedings ahead of the event, and I was often involved in their editing and publication. I have enjoyed attending most meetings. I sadly missed just two, those in Italy, 2011 and in Amsterdam, 2017, due to ill-health. Figure 3 shows the author with Lytton Musselman in the field after the 10th meeting in Turkey in 2009.

## 4. Haustorium

In addition to the major international meetings, there been many more localised or specialised meetings in between, many of them very important and productive, one of particular significance being the *Striga* workshop arranged in Khartoum in 1978 by the International Development Research Center which funded a number of projects on parasitic weeds. It was here that Lytton and I first discussed the idea of a parasitic plants newsletter, resulting in the first issue of ‘Haustorium’ being published in 1979. It started small and had some lapses and problems of funding documented in the item ‘How Haustorium Happens’ in our 50th issue [38], but fortunately, it was able to continue and flourish and has endeavoured to briefly note and summarise as much new literature as was readily available on parasitic plants (not just weeds) twice a year. The mailing list for issue 80 currently approaches 500 from some 60 or more countries. We believe that this newsletter has helped foster the widespread interest in parasitic plants, and extensive international collaboration, which culminated in the establishment of the International Parasitic Plant Society.

## 5. General Publications

In addition to research, my job as ‘Tropical Weeds Liaison Officer’ involved a substantial amount of survey and advisory work across the world, so I had the opportunity to obtain an overview of the parasitic weed problems in many regions, including the Near East [39]. I was also involved in a World Bank project for three years from 1986 to 1989 in Ethiopia, with the remit to establish a *Striga* research project. In addition to producing research papers of local interest, I had the opportunity to survey other parasitic weed problems across the country [40].

And following my formal retirement in 1990, my colleague Charlie Riches and I were able to consolidate our research and survey experience into the book ‘Parasitic Weeds of the World—Biology and Control’ published by CAB International [41]. Since then, I have had nothing better to do than bore you with endless review papers—I apologise. Many of them have been requested, including this one, so it is not all my fault.

## 6. How Do I View the Current Situation?

### 6.1. Striga Species in Cereals

The current extent of the *Striga* problems in maize and sorghum is far from clear. It may be generalised that in high-input farming, there should not be a serious problem with adequate N and P fertilisation and the advanced varieties of sorghum and maize, which should be available locally. In low-input farming, however, the problems persist and may be getting worse locally. The long search for nitrogen fixation in the cereals could one day be an effective solution but seems to be a bit like nuclear fusion for power generation, forever on the horizon.

Research on the strigolactones has been remarkably productive academically, but sadly their use for application to the soil to stimulate suicidal germination has yet to be proved and registered for practical use.

Significant impact has been achieved at least locally in East Africa, using herbicide-resistant varieties combined with herbicide seed treatment [42] but have not been widely adopted. Likewise, The ‘push-pull’ technique using *Desmodium* spp. as intercrops (with or without the herbicide seed treatment) [43] has proved valuable in certain farming systems under the right climatic conditions.

Most recently, the ‘toothpick’ technique, for selective control of *Striga* by a fungal pathogen [44] has been registered for use in East Africa and we look forward to discovering how successful and durable that may prove.

In rice, *Striga* spp. have proved equally intractable, but there has been considerable success with the NERICA varieties developed by the Africa Rice Centre in East Africa. These involve crosses between *Oryza sativa* and the African *O. glaberrima.* These were not developed specifically for *Striga*-resistance but some are proving very effective [45].

In cowpea, *S. gesnerioides*-resistant varieties are available for most areas but, as with *O. cumana* in sunflower, new races continue to occur with extra, or different virulence [46].

Global warming can be expected to result in wider occurrence of *Striga* and other parasitic weed problems [47].

I support the suggestion made elsewhere, for the establishment of a *Striga* (or parasitic weeds) research laboratory—the problems continue to need it.

### 6.2. Other Genera of Orobanchaceae

*Rhamphicarpa fistulosa* continues to be of increasing concern in rice, with limited options for control, which include sowing early maturing varieties, and some partially resistant varieties [48].

For *Alectra vogelii*, resistant cowpea varieties are available) [49], while there are no easy solutions in groundnut/peanut

*Orobanche cumana* in sunflower: (1) reviewed recent progress in the tussle between the breeders and the development of new virulence in sunflower, which I believe the breeders are generally winning. In most regions, resistance is available but the situation keeps changing, keeping the breeders on their toes.

The 2013 workshop in Morocco [50] highlighted the problem from *O. crenata* not only in faba bean, but also in lentil, pea and other legumes across the Mediterranean region, and its serious effects on the nutrition and economy of farmers and countries in the region. In the years since the Malta Symposium there have been many studies on resistance in faba bean. The Egyptian variety Giza 402 was not a productive variety but has been the source of partial resistance in a number of newer varieties. Now a number of varieties are being developed with useful resistance including variety Baraca and derivatives from it, which have shown promise not only for *O. crenata* but also for *O. foetida,* the relatively new problem in Tunisia and Morocco [51].

Meanwhile, *O. crenata* is still spreading. It was first recorded in Ethiopia in 1989, since when it has spread rapidly to many of the important faba-bean growing areas of the country [52].

### 6.3. Other Parasitic Weed Problems

Other parasitic weed problems that I have become familiar with but have done little or no work on include other broomrapes, *O. cernua* parasitising Solanaceae, and the *Phelipanche* species parasitising Solanaceae, Brassicacae and other families. These present serious continuing problems including *P. ramosa* infestations in rape-seed in France [53]. Control of all these depends mainly on herbicides—some treatments are particularly well developed in Israel.

*Cuscuta* spp., especially *C. campestris*, can be severe locally on some crops especially when it is a contaminant of crop seed as in lucerne/alfalfa and in niger seed (*Guizotia abysssinica*), as in Ethiopia. Control depends almost completely on seed-cleaning and on herbicides.

The similar but unrelated *Cassytha filiformis* can be a problem, so far without any developed control measures.

The dwarf mistletoes, *Arceuthobium* species have been described as the most serious disease problem in North American forestry [54]. Their control depends on cultural methods including fire and thinning. Climate change may increase the problem at higher latitudes.

*Viscum album* causes less severe damage, but there are reports of increasing severity of infestation in conifer crops in Europe, to some extent due to drought [55]. There are no established control methods.

## Data Availability

Not applicable.
